# On the Identifiability of Genetic Parameters for Growth in Mice With a Massively Deep Pedigree

**DOI:** 10.1111/jbg.12938

**Published:** 2025-05-02

**Authors:** X. Ding, A. A. Musa, N. Reinsch

**Affiliations:** ^1^ Research Institute for Farm Animal Biology (FBN) Dummerstorf Germany

**Keywords:** data structure, direct genetic effects, identifiability of variance components, inbreeding, maternal genetic effects

## Abstract

In models with direct and maternal genetic effects, structural features of the data are a potential source of bias and low accuracy of estimates for genetic covariance parameters. One of the well‐known reasons for such poor practical identifiability is the lack of dams with own observations. So far, however, no attention has been paid to the impact close relationships may have. Therefore, this genetic‐statistical analysis of growth traits in two unselected mouse lines includes investigations on practical identifiability of genetic (co‐)variances in the light of the observed high levels of co‐ancestry, resulting from massively deep pedigrees. Body weight data had been collected over 33 years (from 1978 to 2011; 145 and 118 generations per line), amounting to approximately 115,000 observations in total for body weight at three developmental stages. Additional analyses of simulated data using the original pedigree structure of one line provided insight into the bias and precision of estimates. Further, closeness to pair‐wise structural non‐identifiability of genetic (co‐)variances was quantified. In univariate analyses, we found genetic correlations between direct and maternal effects all positive for body mass traits at different ages up to mating, except for a single small negative estimate. Overall, multivariate analyses returned somewhat stronger correlations, whereby signs remained unchanged. Simulations showed a tendency toward an upward bias of the direct–maternal genetic correlations and other parameters, especially when the true correlations were higher. For all traits indicators for structural non‐identifiability were narrowly close (> 0.998) to unity, the point at which a pair of covariance components no longer can be identified. This narrowness was stronger for separate partitions of data from later generations with higher average inbreeding and within‐generation co‐ancestry. In conclusion, in models with direct and maternal genetic effects, strong co‐ancestry between parents is another feature of the data structure that may result in bias and inflated standard errors of estimated genetic parameters.

## 1 Introduction

In the context of mixed models that take direct and maternal effects into account, the precision of estimates of their respective genetic (co‐)variances is influenced not only by the absolute and relative size of these genetic co‐variance components but also by certain structural characteristics of the data (Meyer 1992). A critical factor is the presence or absence of phenotypic records for dams, which has been shown to significantly impact the precision and bias of estimates (Gerstmayer 1992; Heydarpour et al. 2008). That issue is particularly relevant for sex‐limited traits in livestock genetics, which are recorded in males only. An example is carcass traits in fattening bulls in dual‐purpose breeds, which are often not available for heifers and cows (Blunk et al. 2016) or have to be considered as different traits in females (Liinamo et al. 2001). Another important structural feature is the number of offspring per dam (Gerstmayer 1992; Heydarpour et al. 2008).

The genetic relationship between a dam's maternal effect and its offspring's direct effect typically is ½ in a base population of unrelated parents (Dickerson 1947; Willham 1972). This relationship has been assumed responsible for causing a confounding between direct and maternal genetic effects and complicating the estimation of genetic parameters. A more general view is that on a linear combination of three different dispersion matrices $G_{i}$ as part of a variance component model including direct and maternal genetic effects. Then each single dispersion matrix $G_{i}$ reflects genetic relationships between animals with observations corresponding to a specific genetic (co‐)variance parameter: $G_{1}=Z_{d}AZ_{d}^{\prime }$ corresponds to the direct genetic variance, $G_{2}=Z_{d}AZ_{m}^{\prime }+Z_{m}AZ_{d}^{\prime }$ to the genetic covariance between direct and maternal effects, and $G_{3}=Z_{m}AZ_{m}^{\prime }$ to the maternal genetic variance ($Z_{i}$ are respective design matrices and A is the numerator‐relationship matrix). A potential similarity between these covariance structures (Gerstmayer 1992) has been assumed as the reason for biased estimates and increased standard errors of genetic (co‐)variances, that is, their poor practical identifiability. The full identity of any pair of dispersion matrices, in contrast, constitutes structural non‐identifiability (Rao and Kleffe 1988), in which case there are many possible values of certain (co‐)variance parameters leading to the same likelihood. Similarities between the aforementioned covariance structures are not routinely quantified in genetic‐statistical analyses, despite their potential role in practical identifiability.

In mice, the relevance of maternal genetic effects on growth traits is well‐documented (Rutledge et al. 1972; Timon and Eisen 1969; Wolf et al. 2011). The proportion of the total phenotypic variance that is due to the maternal genetic variance component tends to decrease with age (Garrick et al. 1989; Van Vleck et al. 1996). Estimates of the genetic correlation between direct and maternal effects in mice vary widely from positive to negative, though this also depends on the specific traits and developmental stages studied (Hanrahan and Eisen 1973; Riska et al. 1985; Robinson et al. 1974). Negative estimates for body weight traits can at least partially be explained by a positive effect of higher body weights on litter size, which in turn has a negative effect on the average birth weight (Falconer and Mackay 2009, 213). The strength of such effects presumably depends on the average mature body weight of a certain strain (Brumby 1960) and may be partially nullified by standardising litter size after birth (Eisen 1973; Rutledge et al. 1972; Timon and Eisen 1969; Willham 1972). In monoparous farm animals, direct‐maternal genetic correlations often have been estimated as negative, such as body weight traits in Australian Hereford and Zebu Cross cattle (Meyer 1992). Small and non‐significant estimates also occasionally were reported (e.g., in Australian Angus cattle; Meyer 1992). Eight country‐specific direct‐maternal correlations for weaning weight in Limousin (Bonifazi et al. 2021) were all negative (the strongest was −0.33) except for Switzerland, with a positive correlation of 0.40. Consistent with this, negative estimates were also reported for body weight traits in sheep (Tosh and Kemp 1994; Ngere et al. 2017) and goats (Latifi et al. 2021). A relevant trait in multiparous farm animals is the birth weight of piglets, for which direct‐maternal correlations are usually reported as negative (Roehe et al. 2010).

Our study utilises data from two mouse lines, DUKssi and DUKb, which have been maintained without any selective breeding as control lines at the Research Institute for Farm Animal Biology in Dummerstorf (Dietl et al. 2004) for over a hundred generations. By employing models that account for direct and maternal genetic effects, we aim for a thorough analysis of genetic parameters for body weight at different ages. Furthermore, we investigate similarities between dispersion matrices, which define the genetic part of the variance component model, by calculating the cosines of their pairwise dot products. For completion, we evaluate potential bias in the estimates and their standard errors in a small simulation study.

## 2 Materials and Methods

### 2.1 Study Animals, Housing and Management

This study involved two non‐selected mouse lines, DUKb and DUKssi (including DUKs and DUKsi sublines), maintained at the Research Institute for Farm Animal Biology (FBN) in Dummerstorf, Germany, between 1978 and their discontinuation in 2011. These lines originated from the FZTDU control line, developed from a hybrid cross of four inbred strains: CBA/Bln, AB/Bln, C57BL/Bln, XVII/Bln, and four outbred strains: NMRI orig., Han: NMRI, CFW, CF1 (Dietl et al. 2004).

The mouse lines were housed under conventional conditions in a semi‐barrier system with open cages, a constant temperature of 22.5°C, controlled humidity, and a 12‐h light/dark cycle. Mice were fed pellets and water *ad libitum*. The pellet composition included 21% protein, 0.4% L‐methionine, 55% starch, 5% sucrose, 5% fat, 5% cellulose, 2% vitamins, and 6% mineral mixture (Altromin No. 1314, Lage, Germany). All animal treatments were approved by the Animal Care Committee of the Ministry of Nutrition, Agriculture, Forestry, and Fishery, Mecklenburg‐Vorpommern, Germany (LVLMV/310‐4/7221.3‐1.1‐018/03).

In each generation, the required number of litters was randomly chosen and about two males and females were sampled from each litter. Males and females then were randomly combined into cages for mating, avoiding mating of full sibs. The number of mating pairs per generation varied between lines and generations. In the DUKb line, there were 50, 100, 80, and 60 mating pairs in generations 1–21, 22–25, 26–95, and from 96 on. The respective numbers in the DUKs subline were 80 and 60 in generations 1–129 and from generation 130 on. The DUKsi subline was branched off from DUKs in generation 78, with 80 mating pairs for the initial 48 generations, then 50 pairs in all subsequent generations. After the mating period of usually 14 days, all males were removed from the mating cages. The size of larger litters was standardised to eight pups in DUKb and nine in DUKssi. At weaning (21 days of age), mice were transferred to Macrolon rearing cages (30 × 12.5 × 12.5 cm). In DUKb, approximately 20 same‐sex individuals from different litters were housed together, while in DUKssi, same‐sex littermates were housed in single cages.

### 2.2 Phenotypic Data and Pedigrees

Body mass was recorded for an average of 2 to 3 randomly selected males per litter at 21 days of age (BM21), at 42 days (BM42) of age, and at mating (BMM). Body mass at mating was the only trait also recorded for females (BMF). The mean age at mating was 62 days in DUKssi. DUKb followed identical mating procedures, with comparable mating ages, although exact dates were not recorded. Table 1 provides descriptive statistics of the data for the DUKb and the DUKssi line. The number of observations was about 8000 for all traits in DUKb. In DUKssi there were more than 15,000 observations per trait, except for BM42 where the number of observations well exceeded 19,000. Mean body mass in males was about 11 g at day 21, and about 28 g at day 42, very much the same as body mass at mating in females, while BMM was higher at 34.5 g in DUKb and 32.7 g in DUKssi. Skewness was small for all traits, indicating nearly symmetric distributions of raw data.

**TABLE 1:** Descriptive statistics for body weight data in mouse lines DUKb and DUKssi by Trait. The traits are: Male body mass at 21 days (BM21), at 42 days (BM42), and at mating (BMM and BMF for males and females, respectively).

Line	Parameters	Trait			
		BM21	BM42	BMM	BMF
DUKb	Number of records, No	8284	8136	7965	7972
	Trait mean ± SD, g	11.25 ± 1.88	28.73 ± 3.24	34.49 ± 3.25	28.40 ± 2.85
	Skewness	−0.05	−0.42	0	0.26
	Kurtosis	0.34	1.41	0.52	0.36
	Age of mother^a^ mean ± SD, days	90.46 ± 3.15	90.46 ± 3.16	90.39 ± 3.20	90.41 ± 3.21
	Litter size mean ± SD, pups	12.51 ± 2.33	12.52 ± 2.33	12.52 ± 2.33	12.53 ± 2.33
	MageDif^b^ mean ± SD, days	—	—	−0.01 ± 0.62	−0.00 ± 0.62
	Generations, No	114	114	113	113
	Litters^c^, No	3311	3278	3229	3242
	Rearing Cages^c^, No	—	504	499	503
DUKssi	Number of records, No	15,119	19,679	15,276	15,299
	Trait mean ± SD, g	10.48 ± 1.89	28.46 ± 3.13	32.67 ± 3.45	27.67 ± 3.16
	Skewness	0.05	−0.41	0.10	0.17
	Kurtosis	0.41	1.21	0.14	−0.14
	Age of mother^d^ mean ± SD, days	84.30 ± 3.53	84.24 ± 3.47	84.41 ± 4.03	84.39 ± 4.03
	Litter size mean ± SD, pups	11.15 ± 2.48	11.30 ± 2.43	11.55 ± 2.31	11.51 ± 2.30
	Mating age mean ± SD, days	—	—	62.03 ± 3.60	62.04 ± 3.60
	Lgen^e^, No	142	182	205	205
	Litters^c^, No	7694	9983	6435	6422

*Note:* —, does not apply.

^a^ Age of mother, age of the mother at farrowing.

^b^ MageDif, mating age difference between individual and generational mean in DUKb.

^c^ Litters, Rearing cages: total number of litters and rearing cages with observations.

^d^ Mating age, age at mating.

^e^ Lgen, generation within subline effects, representing contemporary groups in DUKssi.

**FIGURE 1:**
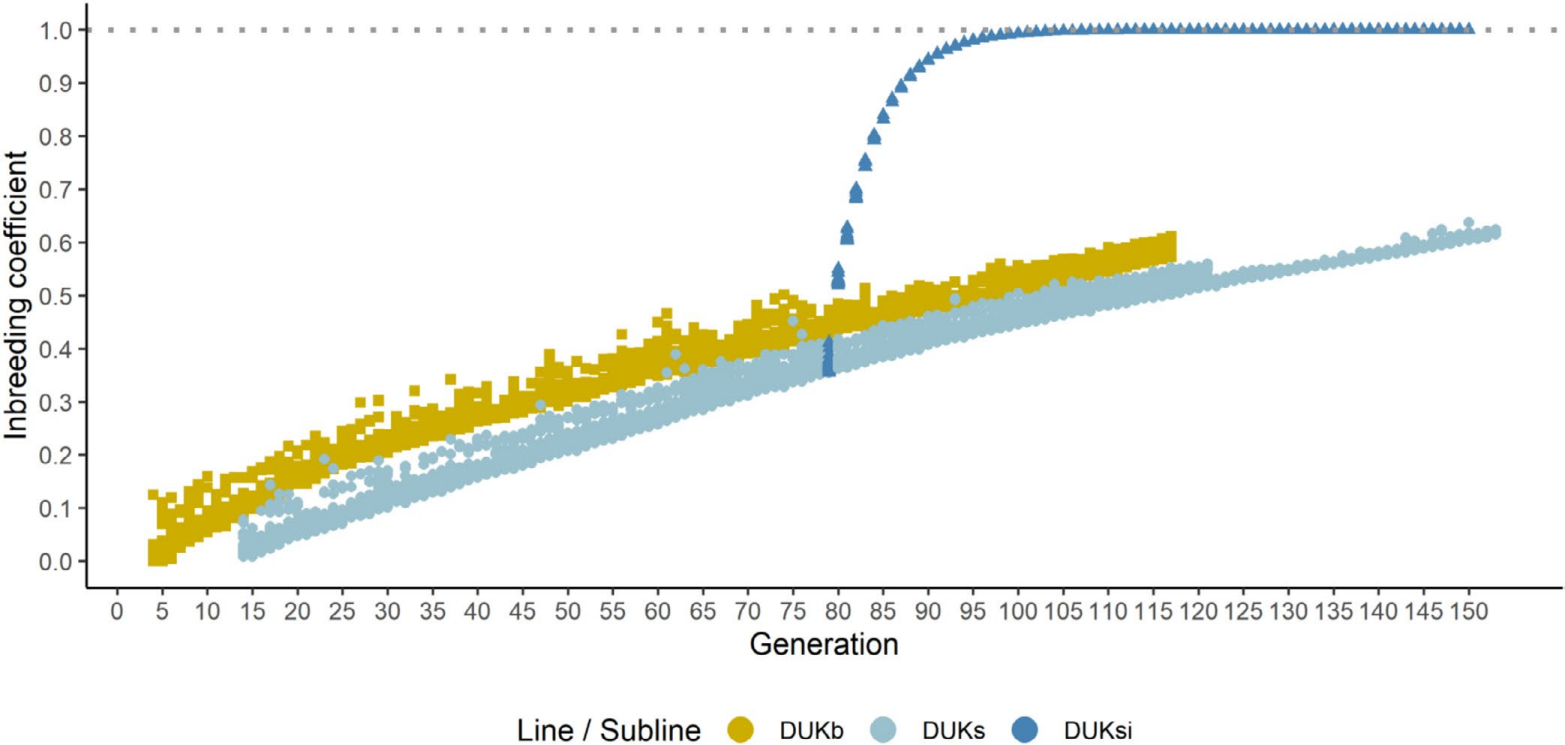
Development of inbreeding over generations in the mouse lines DUKb and DUKssi, the latter a combination of sublines DUKs and DUKsi. Each data point represents the inbreeding coefficient of an individual animal with an own observation for any trait, with zero values excluded. [Colour figure can be viewed at wileyonlinelibrary.com]

Pedigrees were pruned to include only individuals with observations and their ancestors by removing all non‐parents with no phenotypic record from the original pedigrees. Pedigree size after pruning was 16,677 in DUKb and 50,476 in DUKssi. The trajectory of inbreeding is illustrated in Figure 1. The DUKb line and the DUKs subline showed a steady increase in inbreeding from the first generation to the last due to random mating in a closed population. The average inbreeding coefficient for DUKb increased from zero to around 0.61 in the last generation. Similarly, DUKs showed an increase to approximately 0.64. After the DUKsi subline had branched off, inbreeding sharply increased to almost one by generation 90, due to the intentional mating of full sibs. Figure 1 also shows that the variation of inbreeding was limited within generations due to uniform co‐ancestry and lack of population subdivisions within generations. Generation numbers in Figure 1 indicate time intervals of 3 months and include a little delay between generations of both lines.

### 2.3 Statistical Analysis and Genetic Models

#### 2.3.1 Models

Our investigation utilised both single‐trait and multiple‐trait animal models. Restricted maximum likelihood (REML) estimates of (co‐)variance components and derived quantities were obtained by employing the ASReml package (Gilmour et al. 2021). For the sake of comparison, simple animal models with direct genetic effects only were also fitted.

##### 2.3.1.1 Multivariate Direct‐Maternal Animal Models

Animal models included all four body weight traits and were run separately for each line. In matrix notation, the multivariate model equation was:

$$y=\mathrm{Xb}+\mathrm{Zu}+Z_{l}u_{l}+Z_{c}u_{c}+e$$

Thereby y represents the vector of observations ordered by traits and b denotes the vector of fixed effects for all traits. These fixed effects include a generation effect in line DUKb (113 to 114 levels, depending on the trait) and a generation by subline interaction in DUKssi (142, 182, 205, and 205 levels for BM21, BM42, BMM, and BMF, respectively). Age differences between mothers within a generation were taken into account by a linear and quadratic regression on the age of the mother at mating. Likewise, the effect litter size may have on the growth of littermates was corrected for by a linear regression on the number of pups at birth. By definition, BM21 and BM42 were measured at a fixed age, while age varied somewhat at mating, as mating partners were not necessarily born on the same day but put into mating cages on the same date. Models for BMM and BMF included an additional linear regression term specific to each line: for DUKssi, the term represented the age of a candidate with a phenotypic record at mating (directly recorded), while for DUKb, it represented individual birth date deviations from the generational mean (as mating ages were not documented). For means and standard deviations of regressor variables see Table 1.

The first random effect is the vector u, capturing the direct and maternal breeding values for all four traits. The vector $u_{l}$ accounts for common litter environment effects for all traits. As explained above, animals were transferred to rearing cages after weaning. Accordingly, the vector $u_{c}$ represents random common cage environmental effects for post‐weaning traits (excluding BM21) in DUKb. In DUKssi, where same‐sex littermates shared single cages, rearing cage and litter effects were confounded, and their combined effects were captured in $u_{l}$. Finally, the vector e encompasses residuals. Information on factor levels for all traits is summarised in Table 1. Matrices X, Z, $Z_{l}$, and $\;Z_{c}$ are incidence matrices corresponding to the described fixed and random effects. The variance–covariance structure is assumed as follows, with DUKssi lacking $I_{c}⊗C$:

$$\mathrm{Var}\left[\begin{array}{c} u\\ u_{l}\\ u_{c}\\ e \end{array}\right]=\left[\begin{array}{cccc} A⊗G & 0 & 0 & 0\\ 0 & I_{l}⊗L & 0 & 0\\ 0 & 0 & I_{c}⊗C & 0\\ 0 & 0 & 0 & I_{n}⊗R \end{array}\right]$$

$$G=\left[\begin{array}{cc} G_{d} & G_{\mathrm{md}}\\ G_{\mathrm{md}}^{\prime } & G_{m} \end{array}\right]$$

***G*** is the 8 × 8 genetic covariance matrix, where $G_{d}$ is a 4 × 4 submatrix of covariances for direct breeding values, $G_{\mathrm{md}}$ is a 4 × 4 submatrix with covariances between maternal and direct breeding values, $G_{m}$ is a 4 × 4 submatrix with the covariances for maternal breeding values. ***L*** and ***C*** are covariance matrices of the random effects of the common litter environment and the rearing cage. The dimension of ***L*** is 4 × 4 and that of ***C*** is 3 × 3, as there is no rearing cage effect for BM21. R is the 4 × 4 residual covariance matrix with BMF as the only trait recorded in females having zero covariance with the traits recorded in males.

#### 2.3.2 Genetic Parameters of Weight Gain

Variance components for weight gain in the two time intervals between day 21 and day 42 and between day 42 and mating were derived from estimates for body weight traits in males. For distinct kinds of (co‐)variances (direct, maternal, residual, etc.) the respective variance for weight gain ($\sigma_{\text{WGst}}^{2}$) between two time points s and t was calculated as:

$$\sigma_{\text{WGst}}^{2}=\sigma_{\mathrm{BWs}}^{2}+\sigma_{\mathrm{BWt}}^{2}-2\sigma_{\text{BWst}}$$

from the respective estimates, where $\sigma_{\mathrm{BWs}}^{2}$ and $\sigma_{\mathrm{BWt}}^{2}$ are the respective variances for the body weight traits and $\sigma_{\text{BWst}}$ is the covariance.

### 2.4 Heritability and Genetic Correlations

#### 2.4.1 Heritability Estimations

For models with maternal genetic effects the phenotypic variance ($\sigma_{y_{i}}^{2}$) includes the direct genetic variance ($\sigma_{d_{i}}^{2}$), maternal genetic variance ($\sigma_{m_{i}}^{2}$), and their covariance ($\sigma_{{\mathrm{dm}}_{i}}$), in addition to the various non‐genetic environmental variances (litter effect, rearing cage—where applicable, and residual) for trait i:

$$\sigma_{y_{i}}^{2}=\sigma_{d_{i}}^{2}+\sigma_{m_{i}}^{2}+\sigma_{{\mathrm{dm}}_{i}}+\sigma_{l_{i}}^{2}+\sigma_{c_{i}}^{2}+\sigma_{e_{i}}^{2}$$

Then direct heritability ($h_{d_{i}}^{2}$), maternal heritability ($h_{m_{i}}^{2}$), and total heritability ($h_{m_{i}}^{2}$) for each trait was calculated as the ratio $h_{d_{i}}^{2}=\frac{\sigma_{d_{i}}^{2}}{\sigma_{y_{i}}^{2}}$, $h_{m_{i}}^{2}=\frac{\sigma_{m_{i}}^{2}}{\sigma_{y_{i}}^{2}}h_{y_{i}}^{2}=\frac{\sigma_{d_{i}}^{2}+0.5\sigma_{m_{i}}^{2}+1.5\sigma_{{\mathrm{dm}}_{i}}}{\sigma_{y_{i}}^{2}}$, respectively.

### 2.5 Matrix Similarity Analysis

A custom Fortran program was utilised to evaluate the similarity between covariance structures $G_{i}$ as defined in the introduction. The genetic covariance of observations was assumed as a variance component model involving a linear combination of three primary covariance matrices: $Z_{d}AZ_{d}^{\prime }\sigma_{d}^{2}+\left(Z_{d}AZ_{m}^{\prime }+Z_{m}AZ_{d}^{\prime }\right)\sigma_{\mathrm{dm}}+Z_{m}AZ_{m}^{\prime }\sigma_{m}^{2}$ The condition for structural identifiability of the three genetic (co‐)variance parameters is the linear independence of the three covariance matrices involved. This condition is met, if the following 3 × 3 matrix H is of full rank (Rao and Kleffe 1988):

$$H={\left\{\mathrm{tr}G_{i}G_{j}\right\}}_{\mathrm{ij}}$$

where $G_{i}$ and $G_{i}$ represent any pair of the aforementioned covariance structures. Pairwise similarities were then calculated as the dot product‐derived cosine of the angle between two covariance structures (Harville 1997), which can be computed from the elements of H as:

$$r_{\mathrm{uv}}=\frac{\mathrm{tr}\left(G_{i}G_{j}\right)}{\sqrt{\mathrm{tr}\left(G_{i}G_{i}\right)\mathrm{tr}\left(G_{j}G_{j}\right)}}=\frac{\left\langle \left\langle G_{i},G_{j}\right\rangle \right\rangle }{\left\|G_{i}\right\|\left\|G_{j}\right\|}$$

where 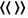 represents the dot product of two matrices, and 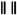 is the norm of a matrix. A cosine of one indicates full linear dependency, meaning the two corresponding covariance components are structurally non‐identifiable. According to these definitions, three pairwise similarities were calculated for each trait and each line. Analyses in the DUKssi line had to be restricted to the DUKs subline, due to the size of the full pedigree.

### 2.6 Simulation of Direct and Maternal Genetic Effects for Validation of Genetic Models

To get more insight into the practical identifiability of genetic (co‐)variance components in terms of bias and standard errors, we conducted simulations using estimated (co‐)variance parameters for each trait in DUKssi and the pedigree from the DUKssi line. Direct and maternal breeding values for the base population, as well as other random effects, were generated as uncorrelated draws from standard normal distributions. These uncorrelated values were then turned into correlated effects at their appropriate scale using a Cholesky decomposition of the genetic covariance matrices. For individuals in later generations, breeding values were calculated by averaging the genetic contributions of parents and adding an uncorrelated Mendelian sampling deviation, thereby taking inbreeding into account. Phenotypic values were then calculated by summing genetic and non‐genetic random effects. For each trait‐specific parameter set, the simulation was repeated one hundred times. REMLestimates of genetic (co‐)variance parameters were then averaged over all hundred repetitions, and bias and standard errors were determined.

### 2.7 Software

The estimation of variance components was conducted using ASReml 4.2 (Gilmour et al. 2021). The matrix similarity analysis was performed using our self‐written Fortran program compiled with GNU Fortran (SUSE Linux) version 7.5.0. All other analyses and pruning of pedigrees were performed with custom scripts written in R version 4.2.2 (R Core Team 2022).

## 3 Results

### 3.1 Genetic Parameters From Univariate Models

Estimates for genetic parameters and derived quantities are shown in Table 2. Direct genetic variances for BM21, BMM, and BMF ranged between 2.98 and 6.45, translating into direct genetic standard deviations between 1.7 and 2.5 g. The direct genetic variance for BM21 was smaller, with a genetic standard deviation in the order of 1 g, that is, 0.7 g in DUKb and 1.1 g in DUKssi. Maternal genetic variances generally were considerably smaller than their direct counterparts, except for BM21, where the maternal genetic component slightly exceeded the direct genetic variance (DUKb) or was almost equal to it (DUKssi). Genetic correlations between direct and maternal effects were all positive, except for BM21 in DUKsi. However, that negative estimate (−0.09) was smaller than its standard error of 0.14. The same was true for two other small positive correlations: BM21 in DUKb and BMM in DUKssi. All other estimates exceeded their respective standard errors, ranging from 0.18 to 0.65. Standard errors for these correlations were very uniform in DUKb and did not exceed 0.16, while standard errors in DUKssi were more variable and ranged from 0.12 to 0.42. The latter presumably is due to the small estimate for the maternal genetic variance of BMF in DUKssi.

**TABLE 2:** Estimates (±standard errors) of genetic parameters from Univariate Analysis for Body Mass Traits by Line. Traits are: Male body mass at 21 days (BM21, g), at 42 days (BM42, g), at mating (BMM, g and BMF, g for males and females respectively). Parameters are: Direct variance ($\sigma_{d}^{2}$), maternal variance ($\sigma_{m}^{2}$), covariance between direct and maternal effects ($\sigma_{\mathrm{dm}}$), correlation between direct and maternal effects ($r_{\mathrm{dm}}$), common litter environmental variance ($\sigma_{l}^{2}$), rearing cage environmental variance ($\sigma_{c}^{2}$), residual variance ($\sigma_{e}^{2}$), total heritability ($h_{t}^{2}$), direct heritability ($h_{d}^{2}$), maternal heritability ($h_{m}^{2}$).

Line	Parameter	Trait			
		BM21	BM42	BMM	BMF
DUKb	$\sigma_{d}^{2}$	0.53 ± 0.11	4.15 ± 0.45	4.26 ± 0.45	5.20 ± 0.49
	$\sigma_{m}^{2}$	1.16 ± 0.19	2.48 ± 0.49	2.05 ± 0.47	0.93 ± 0.28
	$\sigma_{\mathrm{dm}}$	0.08 ± 0.12	0.58 ± 0.39	0.84 ± 0.38	1.07 ± 0.24
	$r_{\mathrm{dm}}$	0.10 ± 0.16	0.18 ± 0.14	0.28 ± 0.16	0.48 ± 0.16
	$\sigma_{l}^{2}$	1.06 ± 0.08	1.03 ± 0.18	0.63 ± 0.18	0.14 ± 0.12
	$\sigma_{c}^{2}$	—	0.15 ± 0.06	0.30 ± 0.07	0.17 ± 0.04
	$\sigma_{e}^{2}$	0.53 ± 0.04	2.85 ± 0.16	3.50 ± 0.17	2.15 ± 0.16
	$h_{t}^{2}$	0.37 ± 0.04	0.56 ± 0.04	0.56 ± 0.03	0.75 ± 0.02
	$h_{d}^{2}$	0.16 ± 0.03	0.37 ± 0.03	0.37 ± 0.03	0.54 ± 0.04
	$h_{m}^{2}$	0.35 ± 0.05	0.22 ± 0.04	0.18 ± 0.04	0.10 ± 0.03
DUKssi	$\sigma_{d}^{2}$	1.20 ± 0.09	2.98 ± 0.23	6.45 ± 0.30	4.04 ± 0.24
	$\sigma_{m}^{2}$	1.02 ± 0.20	0.61 ± 0.24	1.20 ± 0.31	0.18 ± 0.17
	$\sigma_{\mathrm{dm}}$	−0.10 ± 0.17	0.87 ± 0.24	0.17 ± 0.33	0.55 ± 0.16
	$r_{\mathrm{dm}}$	−0.09 ± 0.14	0.64 ± 0.28	0.06 ± 0.12	0.65 ± 0.42
	$\sigma_{l}^{2}$	1.22 ± 0.04	2.20 ± 0.07	1.33 ± 0.09	1.06 ± 0.07
	$\sigma_{e}^{2}$	0.56 ± 0.01	2.26 ± 0.05	2.93 ± 0.08	2.38 ± 0.06
	$h_{t}^{2}$	0.40 ± 0.04	0.51 ± 0.03	0.60 ± 0.03	0.60 ± 0.02
	$h_{d}^{2}$	0.31 ± 0.02	0.33 ± 0.02	0.53 ± 0.02	0.49 ± 0.02
	$h_{m}^{2}$	0.26 ± 0.05	0.07 ± 0.03	0.10 ± 0.03	0.02 ± 0.02

*Note:* —, does not apply.

Estimates of total heritability generally increased with age, from 0.37 at weaning in DUKb (0.40 in DUKssi) up to 0.60 in DUKssi (for both BMM and BMF) and even 0.75 for BMF in DUKb. All estimates for BM42, BMM, and BMF were considerably strong above 0.50, while estimates for BM21 trailed behind that benchmark with a lag of about 0.10. Standard errors for estimates of total heritability were all small in a range between 0.02 and 0.04. For all traits measured after weaning, estimates of direct heritability ranged from 0.33 to 0.54 and often were considerably larger than those for maternal heritability. In contrast, maternal heritability exceeded the direct estimate of BM21, while the direct and maternal estimates were similar in DUKssi.

For the trait BM21, summarising early growth until weaning, variances for the common litter environment showed estimates about twice as large as the residual variance. However, for growth from birth up to ages after weaning, the pattern reversed, with residual variances two to three times the common litter environmental variance, or even more. Though rearing cages were the least important contributor to the phenotypic variance, the inability to estimate a respective variance component in DUKssi may have contributed to the somewhat higher variances for the litter effect, compared to DUKb.

**FIGURE 2:**
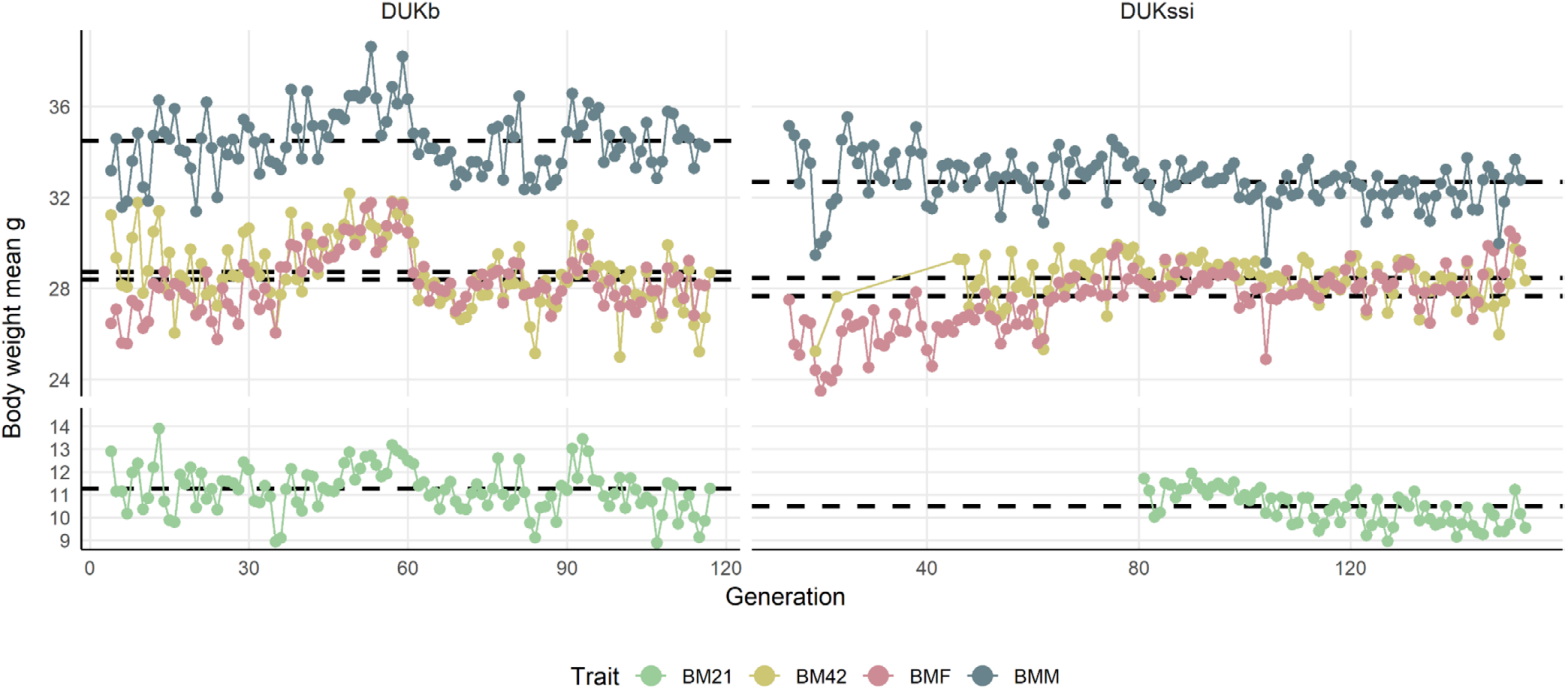
Phenotypic trends across generations for body weight traits in DUKb and DUKssi mouse populations. The figure displays body weight measurements (BM21: 21‐day weight, BM42: 42‐day weight, BMF: female mating weight, BMM: male mating weight). Dots indicate trait specific generational means, while dashed black lines represent trait mean. [Colour figure can be viewed at wileyonlinelibrary.com]

Adding a linear regression on individual inbreeding coefficients to the models led to almost identical estimated covariance components (data not shown). The explanation is that generation effects in our models already account for possible effects of inbreeding through the increase in average inbreeding from generation to generation (Figure 1). Within generations, there remains only limited variation in inbreeding coefficients. Further, phenotypic trends for body weight traits (Figure 2) and litter size (Figure S1) fluctuated around stable baselines over generations with no obvious indication of strong inbreeding depression.

**FIGURE 3:**
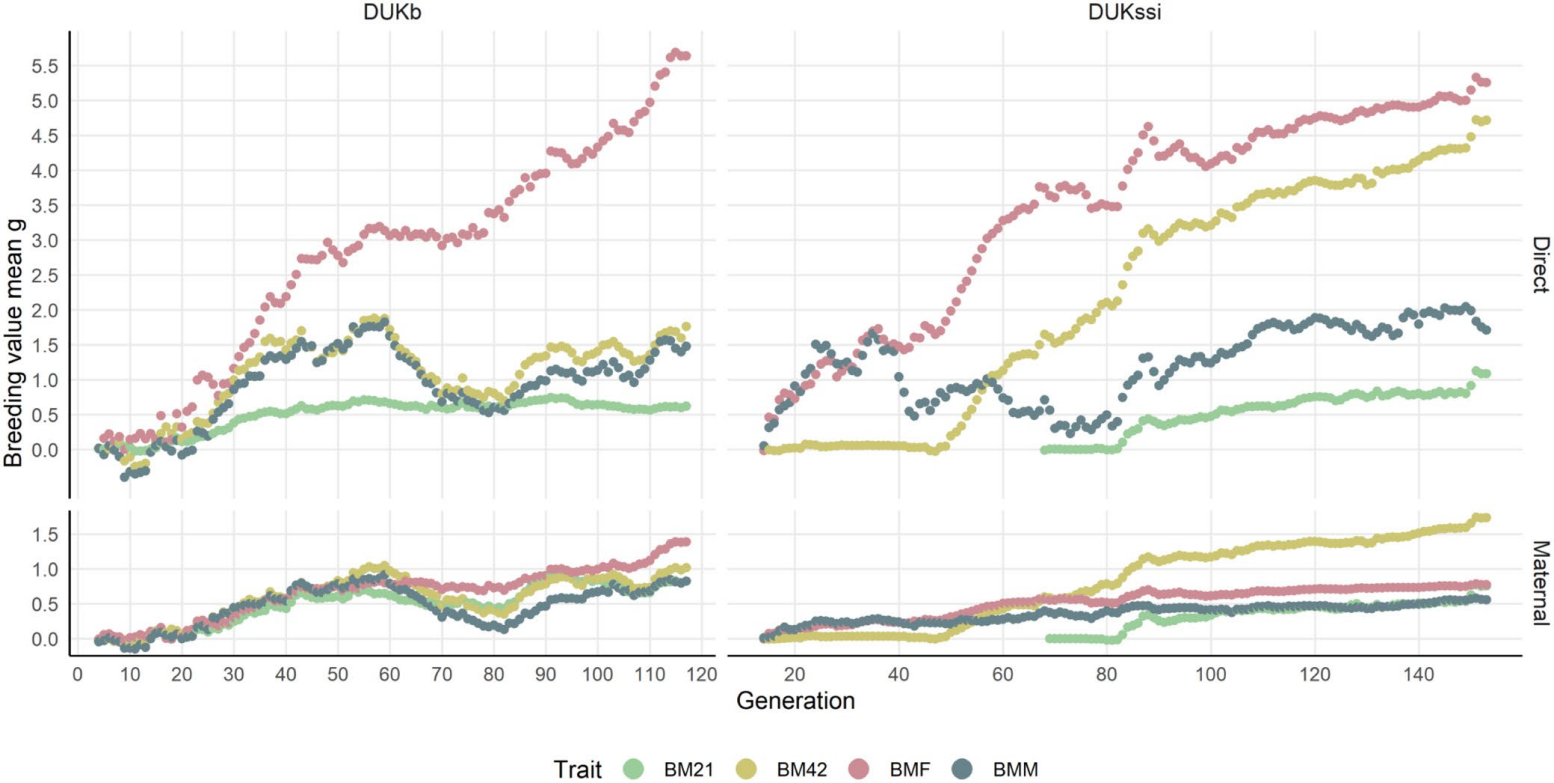
Direct and maternal breeding value mean across generations from univariate analysis for body weight traits in DUKb and DUKssi mouse populations. The figure displays different body weight measurements (BM21: 21‐day weight, BM42: 42‐day weight, BMF: female mating weight, BMM: male mating weight). Note that the genetic trends are tracked up to generation 117 in DUKb and generation 153 in DUKssi populations. [Colour figure can be viewed at wileyonlinelibrary.com]

Direct genetic trends (Figure 3) were found to exist for BMF in both lines and BM42 in DUKb. For these cases, average breeding values increased generation by generation, until they reached a cumulative genetic gain of about 5 g. Other direct genetic trends were small but still positive. Though the underlying reasons cannot be identified, choosing parents from litters without using a random number generator may have contributed through a subjective bias for larger animals. Maternal genetic trends were almost flat (Figure 3).

### 3.2 Multivariate Models: Results for Body Weight Traits

For DUKssi line data we could not obtain convergence of the parameters until the genetic covariance matrix was approximated by a factor analytic model with four factors, which has two covariance parameters less than the full unconstrained genetic covariance matrix of dimension eight by eight. Table 3 gives an overview of the most important results from multivariate analyses. Additional information is provided in Table S2, showing DUKssi results from a fully unconstrained multivariate analysis with some correlations exceeding one.

**TABLE 3:** Estimates (±standard errors) of genetic parameters from multivariate analysis for body mass traits by line. Traits are: Male body mass at 21 days (BM21, g), at 42 days (BM42, g), at mating (BMM, g and BMF, g for males and females respectively). Parameters are: Direct variance ($\sigma_{d}^{2}$), maternal variance ($\sigma_{m}^{2}$), covariance between direct and maternal effects ($\sigma_{\mathrm{dm}}$), correlation between direct and maternal effects ($r_{\mathrm{dm}}$), common litter environmental variance ($\sigma_{l}^{2}$), rearing cage environmental variance ($\sigma_{c}^{2}$), residual variance ($\sigma_{e}^{2}$), total heritability ($h_{t}^{2}$), direct heritability ($h_{d}^{2}$), maternal heritability ($h_{m}^{2}$).

Line	Parameter	Trait			
		BM21	BM42	BMM	BMF
DUKb^a^	$\sigma_{d}^{2}$	0.53 ± 0.10	3.93 ± 0.38	5.26 ± 0.41	5.38 ± 0.40
	$\sigma_{m}^{2}$	1.41 ± 0.18	3.17 ± 0.46	2.96 ± 0.45	0.92 ± 0.24
	$\sigma_{\mathrm{dm}}$	0.19 ± 0.11	0.69 ± 0.34	0.78 ± 0.34	1.01 ± 0.20
	$r_{\mathrm{dm}}$	0.21 ± 0.14	0.20 ± 0.11	0.20 ± 0.10	0.46 ± 0.13
	$\sigma_{l}^{2}$	0.96 ± 0.07	0.91 ± 0.17	0.36 ± 0.16	0.21 ± 0.11
	$\sigma_{c}^{2}$	—	0.15 ± 0.03	0.26 ± 0.05	0.11 ± 0.03
	$\sigma_{e}^{2}$	0.53 ± 0.03	2.95 ± 0.14	3.25 ± 0.15	2.06 ± 0.14
	$h_{t}^{2}$	0.42 ± 0.03	0.56 ± 0.03	0.61 ± 0.03	0.76 ± 0.02
	$h_{d}^{2}$	0.15 ± 0.03	0.33 ± 0.03	0.41 ± 0.03	0.56 ± 0.03
	$h_{m}^{2}$	0.39 ± 0.04	0.27 ± 0.04	0.23 ± 0.03	0.09 ± 0.02
DUKssi^b^	$\sigma_{d}^{2}$	0.96 ± 0.07	2.99 ± 0.16	6.33 ± 0.25	4.28 ± 0.20
	$\sigma_{m}^{2}$	1.41 ± 0.14	1.17 ± 0.15	1.42 ± 0.22	0.15 ± 0.06
	$\sigma_{\mathrm{dm}}$	−0.20 ± 0.09	1.07 ± 0.11	0.50 ± 0.21	0.63 ± 0.10
	$r_{\mathrm{dm}}$	−0.17 ± 0.07	0.57 ± 0.07	0.17 ± 0.08	0.79 ± 0.14
	$\sigma_{l}^{2}$	1.15 ± 0.03	2.02 ± 0.07	1.38 ± 0.07	1.11 ± 0.06
	$\sigma_{e}^{2}$	0.58 ± 0.01	2.23 ± 0.05	2.91 ± 0.07	2.33 ± 0.06
	$h_{t}^{2}$	0.35 ± 0.02	0.55 ± 0.01	0.62 ± 0.02	0.62 ± 0.01
	$h_{d}^{2}$	0.25 ± 0.02	0.32 ± 0.01	0.50 ± 0.02	0.50 ± 0.02
	$h_{m}^{2}$	0.36 ± 0.03	0.12 ± 0.02	0.11 ± 0.02	0.02 ± 0.01

*Note:* —, does not apply.

^a^ DUKb, four‐trait unstructured model with direct and maternal genetic effects.

^b^ DUKssi, four‐trait genetic covariances from factor analytic model with 4 factors.

Estimates for direct genetic variances were quite stable in comparison to results from univariate analyses. When expressed in terms of genetic standard deviations, the largest drop of a single value was −0.12 g (BM21 in DUKssi), and the largest increase was of about the same size at 0.23 g (BMM in DUKb).

Maternal genetic variances for the male traits BM21, BM42, and BMM were all estimated somewhat higher in our multivariate analyses, whereby the change in terms of genetic standard deviations never exceeded 0.29 g (BMM in DIKb). For BMF, multivariate estimates for the maternal genetic variance were essentially equal to univariate results in both lines.

Estimated correlations between genetic direct and maternal effects from multivariate analyses showed the same pattern of signs as in our single‐trait analyses: all correlations were positive, except for BM21 in DUKssi. For four traits, the multivariate estimates remained very weak (absolute value smaller than 0.19) or weak, respectively; for traits BM21 and BM42 in DUKb; and BM21 and BMM in DUKssi. Likewise, the moderate correlation for BMF in DUKb remained almost constant, whereas the multivariate estimate of the same correlation for BM42 in DUKssi dropped somewhat to 0.57 (from 0.64). The already strong univariate correlation estimate of 0.65 for BMF in DUKssi was even higher at 0.79 in the multivariate analysis. No multivariate estimate, be it weak or strong, was, however, smaller than its standard error in the multi‐trait analyses.

Estimated variance components for non‐genetic effects also were very similar to univariate models when derived from multiple‐trait models (Table 3).

### 3.3 Parameters for Weight Gain

Transforming variance components for body mass traits into parameters for weight gain (Table 4) resulted in estimates for total and direct heritability that were quite similar. Values for early post‐weaning gain from day 21 to day 42 were larger by about 0.20 (ranging from 0.39 to 0.54) compared to gain from day 42 to mating (ranging from 0.19 to 0.26). In contrast, estimates for maternal heritability were considerably lower (between 0.06 and 0.17). Genetic correlations between direct and maternal genetic effects on weight gain all were negative, displaying as very weak in DUKb and moderate to strong in DUKssi.

**TABLE 4:** Heritability and Correlations between Direct and Maternal Genetic Effects on Weight Gain Traits by Line. Traits are: Weight gain from day 21 to day 42 (BM21‐42, g) and weight gain from day 42 to mating day (BM42‐MM, g). Parameters are: Total heritability ($h_{t}^{2}$), direct heritability ($h_{d}^{2}$), maternal heritability ($h_{m}^{2}$). Standard errors in brackets.

Line	Parameter	Trait	
		BM21‐42	BM42‐MM
DUKb	$h_{t}^{2}$	0.41 ± 0.03	0.25 ± 0.03
	$h_{d}^{2}$	0.39 ± 0.04	0.21 ± 0.04
	$h_{m}^{2}$	0.06 ± 0.03	0.10 ± 0.03
	$r_{\mathrm{dm}}$	−0.02 ± 0.21	−0.07 ± 0.21
DUKssi	$h_{t}^{2}$	0.48 ± 0.04	0.19 ± 0.08
	$h_{d}^{2}$	0.54 ± 0.03	0.26 ± 0.10
	$h_{m}^{2}$	0.17 ± 0.04	0.07 ± 0.09
	$r_{\mathrm{dm}}$	−0.33 ± 0.10	−0.50 ± 0.43

### 3.4 Similarity Between Covariance Structures and Simulation Results

Cosines, as defined by pairwise dot products between (co‐)variance parameter‐specific covariance matrices, are presented in Table 5. All values were extraordinarily close to unity, the value at which a pair of respective (co‐)variances becomes structurally non‐identifiable. All values exceeded 0.999, with any differences only visible in the last two of five digits and not exceeding 0.00035. The matrix H was of full rank for all traits and in both lines with the smallest eigenvalue always clearly positive in the thousands, with a single exception at 258 (BM21 in DUKs). In all cases, however, the largest eigenvalue exceeded the smallest by several magnitudes (data not shown).

**TABLE 5:** Pairwise cosine similarity values between dispersion matrices for genetic covariance parameters by trait. Traits are: Male body mass at 21 days (BM21, g), at 42 days (BM42, g), at mating (BMM, g and BMF, g for males and females respectively).

Line	Cosine	Trait			
		BM21	BM42	BMM	BMF
DUKb	1–2	0.99988	0.99988	0.99987	0.99989
	2–3	0.99985	0.99984	0.99983	0.99983
	1–3	0.99965	0.99965	0.99963	0.99965
DUKs	1–2	0.99998	0.99996	0.99990	0.99991
	2–3	0.99996	0.99994	0.99986	0.99986
	1–3	0.99993	0.99988	0.99970	0.99973

*Note:* Cosine similarity between $Z_{d}{\mathrm{AZ}}_{d}^{'}$ and $\left(Z_{d}{\mathrm{AZ}}_{m}^{'}+Z_{m}{\mathrm{AZ}}_{d}^{'}\right)$; between $\left(Z_{d}{\mathrm{AZ}}_{m}^{'}+Z_{m}{\mathrm{AZ}}_{d}^{'}\right)$ and $Z_{m}{\mathrm{AZ}}_{m}^{'}\sigma_{m}^{2}$; between $Z_{d}{\mathrm{AZ}}_{d}^{'}$ and $Z_{m}{\mathrm{AZ}}_{m}^{'}$ are abbreviated as 1–2, 2–3, 1–3, respectively.

The simulation study (Table 6) examined the consistency of REML estimates of genetic parameters: when the original REML estimates indicated weak direct‐maternal relationships (BM21: $r_{\mathrm{dm}}$ = −0.09; BMM: $r_{\mathrm{dm}}$ = 0.06), the mean estimates from one hundred simulated datasets were less biased. For BM21, the bias relative to original estimates was negligible for direct variance (bias = −0.02, SD = 0.08) and maternal variance (bias = 0.03, SD = 0.21). BMM showed similarly small bias for both direct (bias = 0.02, SD = 0.29) and maternal (bias = 0.03, SD = 0.33) variance estimates. However, for traits where the original REML estimates indicated stronger direct‐maternal relationships (BM42: $r_{\mathrm{dm}}$ = 0.64; BMF: $r_{\mathrm{dm}}$ = 0.65), larger and strongly significant biases emerged between original and simulation‐based estimates. In BM42, simulation‐based estimates showed downward bias for direct variance (bias = −1.19, SD = 0.21) and upward bias for maternal variance (bias = 1.25, SD = 0.31) compared to the original estimates. BMF displayed similar patterns, with a downward bias in direct variance (bias = −1.52, SD = 0.29) and an upward bias in maternal variance (bias = 1.55, SD = 0.36). These biases were consistent across repetitions. The results suggest that, with the given structure of pedigree and number of observations, REML estimation yields more biased results when true direct‐maternal relationships are stronger.

**TABLE 6:** Summary of simulation results for line DUKssi using univariate animal model with maternal effects, 100 repetitions per trait. Traits are: Male body mass at 21 days (BM21, g), at 42 days (BM42, g), at mating (BMM, g and BMF, g for males and females respectively). Parameters are: Direct variance ($\sigma_{d}^{2}$), maternal variance ($\sigma_{m}^{2}$), covariance between direct and maternal effects ($\sigma_{\mathrm{dm}}$), correlation between direct and maternal effects ($r_{\mathrm{dm}}$), first eigenvalue of genetic covariance matrix (Eigen).

Trait	Parameter	True value	Mean	Bias	SD	*p* ^a^
BM21	$\sigma_{d}^{2}$	1.20	1.19	−0.02	0.08	< 0.05
	$\sigma_{m}^{2}$	1.03	1.05	0.03	0.21	0.16
	$\sigma_{\mathrm{dm}}$	−0.10	−0.11	0.01	0.18	0.71
	$r_{\mathrm{dm}}$	−0.09	−0.09	0.01	0.16	0.64
	Eigen	1.25	1.34	0.09	0.18	< 0.001
BM42	$\sigma_{d}^{2}$	2.98	1.79	−1.19	0.21	< 0.001
	$\sigma_{m}^{2}$	0.61	1.86	1.25	0.31	< 0.001
	$\sigma_{\mathrm{dm}}$	0.87	1.40	0.53	0.20	< 0.001
	$r_{\mathrm{dm}}$	0.64	0.78	0.14	0.14	< 0.001
	Eigen	3.26	3.24	−0.02	0.18	0.28
BMM	$\sigma_{d}^{2}$	6.45	6.47	0.02	0.29	0.55
	$\sigma_{m}^{2}$	1.20	1.23	0.03	0.33	0.42
	$\sigma_{\mathrm{dm}}$	0.17	0.39	0.22	0.34	< 0.001
	$r_{\mathrm{dm}}$	0.06	0.16	0.10	0.14	< 0.001
	Eigen	6.45	6.51	0.06	0.28	0.03
BMF	$\sigma_{d}^{2}$	4.04	2.53	−1.52	0.29	< 0.001
	$\sigma_{m}^{2}$	0.18	1.73	1.55	0.36	< 0.001
	$\sigma_{\mathrm{dm}}$	0.55	1.90	1.35	0.12	< 0.001
	$r_{\mathrm{dm}}$	0.65	0.93	0.28	0.07	< 0.001
	Eigen	4.12	4.09	−0.02	0.17	0.15

^a^ *p*‐value, *t*‐test for non‐zero bias, based on a sample size of 100.

## 4 Discussion

The use of mixed models for the analysis of selection experiments became common practice during the 80ies of the last century (Sorensen and Kennedy 1984). Such experiments frequently include data from unselected control lines, in our case constituting the only data source. As stressed by Sorensen and Kennedy (1984), the numerator‐relationship matrix accounts for the decline in variance due to genetic drift—undoubtedly an essential property when the number of generations is on the magnitude of one hundred. Many of the earlier adopters of mixed models in that realm, however, did not introduce maternal genetic effects in their analyses of growth traits in mice. Examples are investigations of the change of genetic parameters for six‐week body weight (Heath et al. 1995) and lean mass in 10‐week‐old mice (Beniwal et al. 1992a) after twenty generations of selection as well as after an additional 18 generations (Beniwal et al. 1992b). This is in stark contrast to results from a plethora of earlier cross‐fostering experiments (Bateman 1954; Cox et al. 1959), which provided evidence for the significance of maternal effects on the early growth of mice. Finally, Wolf et al. (2011) successfully mapped QTL for maternal genetic effects on mouse growth. Therefore, the statistical significance of maternal genetic effects in our data (for error probabilities see Table S1) did not come as a surprise.

For the DUKb mouse line, maternal genetic heritability occasionally surpasses direct genetic heritability, particularly in early stages (e.g., BM21), aligning with previous studies that suggest neglecting maternal effects may lead to an inflation of direct genetic contributions (Albuquerque and Meyer 2001; Cowley et al. 1989; Wolf et al. 2011). This confirms the conclusion (Rutledge et al. 1972) that maternal genetic variance is more prominent at younger ages and continues to be influential in later stages.

Less clear is the overall picture concerning the sign and strength of the genetic covariance/correlation between direct and maternal effects on body weight and weight gain. This covariance for body weight at 12 days of age has been estimated as negative in a classical study using least squares (Robinson et al. 1974); its size was classified as a small contribution to the phenotypic variance and translates into a medium negative correlation of −0.49. This study covered data from nine generations and two lines, yet the number of animals did not exceed 2000, judged by the reported numbers of families per generation. In contrast, Eisen et al. (1970) found a small but positive covariance for the same trait. Further, Hanrahan and Eisen (1973) reported a consistent antagonism between direct and maternal effects on body weight traits and weight gain. Their study comprised around 5000 animals in 14 generations. Negative correlations were reported as very strong at 6 weeks of age, medium at 8 weeks, and very weak for gain from 6 to 8 weeks.

Our analyses resulted in positive estimates (with one exception), some of which were small. That is in agreement with cross‐fostering experiments (Riska et al. 1985; Swartz and Famula 1994), from which one could expect positive correlations. From our small simulation study, it can be deduced that the larger positive estimates likely are somewhat biased in an upward direction. Standardising litter size is a common practice in selection experiments and studies of maternal effects in laboratory species (Legates 1972) and reduces the effects of larger litters leading to small mothers in the next generation, potentially with poorer maternal abilities. How much variation is removed by standardisation of litter size may also depend on the interplay between the chosen maximum number of pups and the average body size of mothers, as smaller strains provide a poorer maternal performance (Brumby 1960). In conclusion, as mouse lines differ in their body weight, positively estimated genetic correlations may beline‐specific and valid only for the chosen standard litter size.

The high pair‐wise similarity between dispersion matrices in our mouse lines is driven by a high level of co‐ancestry and inbreeding. As shown in Table S3, the similarity between dispersion matrices increases with the depth of the pedigree, when observations were taken from consecutive 20‐generation intervals. This can be further confirmed by constructing small example data sets (data not shown) with varying co‐ancestry. Simulations demonstrated, however, that estimates of genetic covariance parameters may be unbiased, even though pair‐wise similarities between dispersion matrices were at the brink of structural non‐identifiability. The bias of estimates was dependent on the true parameters. With a higher true correlation between direct and maternal genetic effects, more data may be necessary to compensate for otherwise poor practical identifiability. Any effort in generating more data by adding extra generations to a line with an already high level of co‐ancestry will, however, be limited compared to the same amount of observations from, for example, a repeated line with a common pedigree. There is no obvious remedy for poor practical identifiability once the data have been generated. In planned trials, experimenters could choose a favourable design such as generating maternal half‐sib families (Maiorano et al. 2019). Any positive effect of such a design is, however, likely to be overridden by very strong co‐ancestry, just like the effect of dams with own phenotype (BWF) made no difference in our data.

Quantifying pair‐wise similarities of dispersion matrices in models with several genetic effects per trait should probably become a more common practice. Analyses of the profile‐likelihood for certain parameters have been suggested (Wieland et al. 2021) in other areas of application to assess the practical identifyability of certain parameters of interest. For many quantitative‐genetic studies, this seems to be too demanding computationally. Computing pair‐wise similarities from the elements of the H matrix as well as an inspection of the eigenvalues of that matrix may serve as an indicator for potential problems with practical identifiability. The size of the numerator‐relationship matrix of the complete pedigree will possibly exceed the available computer memory in many applications. If this cannot be overcome by improved software, a characteristic subset of the data may be a good substitute.

## 5 Conclusion

Our study has identified close relationships between parents and high levels of inbreeding as another structural feature with a potentially detrimental effect on the practical identifiability of genetic covariance components in models with direct and maternal genetic effects. This may result in biased estimates and elevated standard errors. Whether such effects are relevant for a given data set depends, however, also on the actual size of the unknown parameters. As a general diagnostic measure of practical identifiability, we recommend the computing of pair‐wise similarities between genetic dispersion matrices that define the underlying variance component model.

## Author Contributions

X. Ding performed all statistical analyses, interpreted the results, and drafted the manuscript. A. A. Musa conducted the simulations, interpreted the results, and revised the manuscript. N. Reinsch conceived and supervised the study, contributed to the design of the similarity study, drafted the Fortran program, interpreted the results, and critically revised the manuscript. All authors read and approved the final manuscript.

## Conflicts of Interest

The authors declare no conflicts of interest.

## Supporting information


Appendix S1.

## Data Availability

Genetic data are available from the corresponding author upon request.
