# Supplementary material for: On the Identifiability of Genetic Parameters for Growth in Mice With a Massively Deep Pedigree
Source: J Anim Breed Genet. 2025 May 2;142(6):706–17. doi: 10.1111/jbg.12938 (PMC12501679; doi:10.1111/jbg.12938)
Supplement: Supplementary file 1 — Appendix S1. [file JBG-142-706-s001.docx]

# Supplementary file

**Tables**

**TABLE S1.** Log-likelihood ratio test for univariate models with and without maternal effects

|  | DUKb | | |  | DUKssi | | |
| --- | --- | --- | --- | --- | --- | --- | --- |
| Trait | $Logl_{UD}$^a^ | $Logl_{UDM}$^b^ | P value |  | $Logl_{UD}$^a^ | $Logl_{UDM}$^b^ | P value |
| BM21 | -6126.24 | -6072.14 | $3.20\times10^{-24}$ |  | -11741.30 | -11702.30 | $1.15\times10^{-17}$ |
| BM42 | -11781.63 | -11734.40 | $3.10\times10^{-21}$ |  | -25961.91 | -25928.60 | $3.42\times10^{-15}$ |
| BMM | -11876.10 | -11829.80 | $7.80\times10^{-21}$ |  | -21704.97 | -21685.32 | $2.92\times10^{-09}$ |
| BMF | -10628.60 | -10600.10 | $4.20\times10^{-13}$ |  | -19448.00 | -19437.66 | $3.23\times10^{-05}$ |

All tests have 2 degrees of freedom.

^a^$Logl_{UD}$, log-likelihood from the univariate model without maternal effects;

^b^$Logl_{UDM}$, log-likelihood from the univariate model with maternal effects.

**TABLE S2**. Unrestrained **Estimates of genetic Parameters from Multivariate Analyses for Body Mass Traits in DUKssi mice,** . Traits are: male body mass at 21 days (BM21, g), at 42 days (BM42, g), at mating (BMM, g and BMF, g for males and females respectively). Parameters are: direct variance ($\sigma_{d}^{2}$), maternal variance ($\sigma_{m}^{2}$), covariance between direct and maternal effects ($\sigma_{dm}$), correlation between direct and maternal effects ($r_{dm}$), common litter environmental variance ($\sigma_{pm}^{2}$), residual variance ($\sigma_{e}^{2}$), total heritability ($h_{t}^{2}$), direct heritability ($h_{d}^{2}$), maternal heritability ($h_{m}^{2}$ ).

|  | Trait | | | |
| --- | --- | --- | --- | --- |
| Parameter | BM21 | BM42 | BMM | BMF |
| $\sigma_{d}^{2}$ | 0.98±0.08 | 2.94±0.20 | 6.42±0.27 | 4.28±0.22 |
| $\sigma_{m}^{2}$ | 1.45±0.19 | 0.98±0.21 | 1.33±0.27 | 0.15±0.16 |
| $\sigma_{dm}$ | -0.25±0.14 | 1.24±0.20 | 0.55±0.26 | 0.64±0.14 |
| $r_{dm}$ | -0.21±0.11 | 0.73±0.18 | 0.19±0.10 | 0.80±0.52 |
| $\sigma_{pm}^{2}$ | 1.15±0.03 | 2.04±0.07 | 1.40±0.08 | 1.12±0.06 |
| $\sigma_{e}^{2}$ | 0.57±0.01 | 2.24±0.05 | 2.89±0.07 | 2.33±0.06 |
| $h_{t}^{2}$ | 0.34±0.04 | 0.56±0.02 | 0.63±0.02 | 0.62±0.02 |
| $h_{d}^{2}$ | 0.25±0.02 | 0.31±0.02 | 0.51±0.02 | 0.50±0.02 |
| $h_{m}^{2}$ | 0.37±0.04 | 0.10±0.02 | 0.11±0.02 | 0.02±0.02 |

**TABLE S3.** **Cosine Similarity Values between Dispersion Matrices for Genetic Covariance Parameters by 20 Generations Interval.** Generation range, observations are male body mass at 21 day (BM21, g) for DUKb began at 4th generation after data pruning.

|  | Generation range | | | |
| --- | --- | --- | --- | --- |
| Cosine | 4-23 | 24-43 | 44-63 | 64-83 |
| 1-2 | 0.99107 | 0.99896 | 0.99956 | 0.99979 |
| 2-3 | 0.98806 | 0.99858 | 0.99940 | 0.99971 |
| 1-3 | 0.97857 | 0.99744 | 0.99889 | 0.99946 |

Cosine similarity between$Z_{d}AZ_{d}^{'}$and$(Z_{d}AZ_{m}^{'}+Z_{m}AZ_{d}^{'})$; between$(Z_{d}AZ_{m}^{'}+Z_{m}AZ_{d}^{'})$and$Z_{m}AZ_{m}^{'}\sigma_{m}^{2}$; between $Z_{d}AZ_{d}^{'}$and$Z_{m}AZ_{m}^{'}$are abbreviated as 1-2, 2-3, 1-3, respectively.

**Figures**


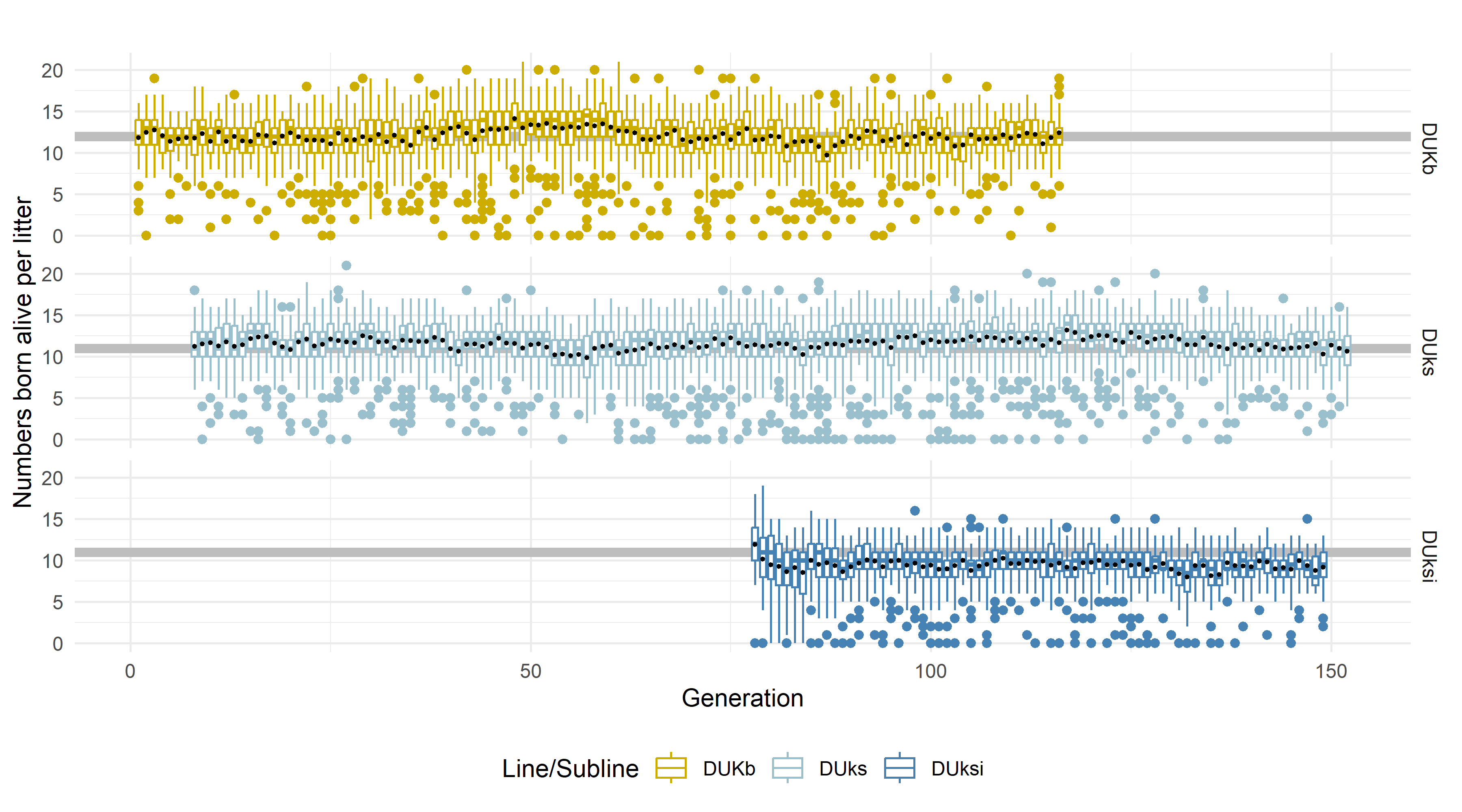


**FIGURE S1. Number of pups born alive per litter across generations in mouse populations.** Black dots indicate generational means, while coloured boxplots represent their distributions within each generation. Grey lines represent the line specific overall mean.
